# Supplementary material for: Content-rich biological network constructed by mining PubMed abstracts
Source: BMC Bioinformatics. 2004 Oct 8;5:147. doi: 10.1186/1471-2105-5-147 (PMC528731; doi:10.1186/1471-2105-5-147)
Supplement: Additional File 5 — The original Chilibot query results of the term "long-term potentiation (LTP)" and 22 other terms, limiting the latest references analyzed to the years 1990, 1995, 2000, and 2004. [file 1471-2105-5-147-S5.bz2 › chilibotAdditionalFile5/ltp2000/html/AMPA_ACTIN.html]

 


 **AMPA** and **ACTIN** 
  
Found 17 abstracts in PubMed,  **17 abstracts were retrieved and analyzed**.  


---

 Search Google  |
 PDF files only 
|  EDU domain only 

---

**Interactive relationship** (e.g. stimulation, inhibition, etc)

- Here we show that  **actin**  dynamics in spines are potently inhibited by activation of either  **AMPA**  or NMDA subtype glutamate receptors.  Ref: 10966619 Nat Neurosci, 2000
- Moreover, disruption of  **actin**  filaments in cultured cortical neurons dramatically reduced the level of surface  **AMPA**  receptors.  Ref: 11050113 J Neurosci, 2000
- Depolymerization of F  **actin**  led to a 40% decrease in both the number of synaptic NMDA receptor NMDAR1 clusters and the number of  **AMPA**  receptor GluR1 labeled spines.  Ref: 9502803 J Neurosci, 1998

- :-)

  **Parallel relationship** (e.g. studied together, co-existance, homology, etc.)

  - Role of  **actin**  in anchoring postsynaptic receptors in cultured hippocampal neurons differential attachment of NMDA versus  **AMPA**  receptors.  Ref: 9502803 J Neurosci, 1998
  - This selectively solubilized  **actin**  and spectrin while other prominent PSD proteins, such as CaMK II alpha, the  **AMPA**  and NMDA type glutamate receptors and GABA receptors, were not extracted at all.  Ref: 9037539 Brain Res Mol Brain Res, 1996
  - We used  **actin**  perturbing agents and detergent extraction of primary hippocampal cultures to test directly the role of the  **actin**  cytoskeleton in localizing GABAA receptors,  **AMPA**  and NMDA type glutamate receptors, and potential anchoring proteins at postsynaptic sites.  Ref: 9502803 J Neurosci, 1998
  - We constructed recombinant adenoviruses encoding rat  **AMPA**  receptor subunit cDNAs, GluR1 AxCAGluR1 and silently mutated GluR2 AxCAGluR2X with modified chicken beta  **actin**  promoter and cytomegalovirus immediate early enhancer.  Ref: 9406922 Brain Res Mol Brain Res, 1997
  - These results suggest that protein 4.1N may link  **AMPA**  receptors to the  **actin**  cytoskeleton.  Ref: 11050113 J Neurosci, 2000
